# Impacts of diurnal temperature and larval density on aquatic development of *Aedes aegypti*

**DOI:** 10.1371/journal.pone.0194025

**Published:** 2018-03-07

**Authors:** Josef Zapletal, Madhav Erraguntla, Zach N. Adelman, Kevin M. Myles, Mark A. Lawley

**Affiliations:** 1 Department of Industrial and Systems Engineering, Texas A&M University, College Station, Texas, United States of America; 2 Department of Entomology, Texas A&M University, College Station, Texas, United States of America; University of Texas Medical Branch, UNITED STATES

## Abstract

The increasing range of *Aedes aegypti*, vector for Zika, dengue, chikungunya, and other viruses, has brought attention to the need to understand the population and transmission dynamics of this mosquito. It is well understood that environmental factors and breeding site characteristics play a role in organismal development and the potential to transmit pathogens. In this study, we observe the impact of larval density in combination with diurnal temperature on the time to pupation, emergence, and mortality of *Aedes aegypti*. Experiments were conducted at two diurnal temperature ranges based on 10 years of historical temperatures of Houston, Texas (21–32°C and 26.5–37.5°C). Experiments at constant temperatures (26.5°C, 32°C) were also conducted for comparison. At each temperature setting, five larval densities were observed (0.2, 1, 2, 4, 5 larvae per mL of water). Data collected shows significant differences in time to first pupation, time of first emergence, maximum rate of pupation, time of maximum rate of pupation, maximum rate of emergence, time of maximum rate of emergence, final average proportion of adult emergence, and average proportion of larval mortality. Further, data indicates a significant interactive effect between temperature fluctuation and larval density on these measures. Thus, wild population estimates should account for temperature fluctuations, larval density, and their interaction in low-volume containers.

## Introduction

Recent outbreaks caused by vector borne pathogens including those caused by Zika, dengue and chikungunya viruses have highlighted the need for understanding the factors influencing the development of mosquitoes and their ability to transmit disease agents. *Aedes aegypti* are well known to be competent vectors of these viruses and flourish in warm and temperate climates [1–5]. Breeding in man-made containers (e.g. tires, flower pots, garbage etc.) and primarily feeding on humans, *Ae*. *aegypti* thrive in proximity to humans [6,7]. With increasing temperatures expanding the suitable range of *Ae*. *aegypti*, the threat of disease transmission has expanded into new geographical areas [8–10].

Environmental conditions have been shown to impact the development of *Aedes* mosquitoes. Temperature has been shown to be a significant factor in aquatic development, with warmer temperatures resulting in a shorter development time [11–15]. Light exposure has been shown to impact the rate of development, where longer light exposure results in shorter development times [16–18]. The amount of food available has also been shown to have an impact on the aquatic development time, with higher levels of nutrients resulting in reduced competition and shorter development times [13,19,20]. Larval density plays a significant role due to the release of juvenile hormone by larvae and competition for limited food resources in container habitats; higher larval densities can delay or prevent pupation [13,21–25]. Previous efforts to understand the effects of larval density on the development time of *Ae*. *aegypti* have been conducted under constant temperatures [25]. These studies did not address the effects of natural diurnal temperature fluctuations and interaction between larval densities and diurnal temperatures. Previous studies have shown that diurnal temperature conditions have significant impact on the life history traits of *Ae*. *aegypti* when compared to constant temperatures—lengthening the time to adult emergence, increasing larval mortality, reducing adult female reproductive output, reducing adult body size, and increasing vector competence [26–28]. Lambrechts et al. (2011) focused on temperature fluctuations at thermal extremes, but did not address the interactions between the larval densities and temperature fluctuations [29].

The goal of this work is to analyze the combined effects of diurnal temperature and larval density on the aquatic development and mortality of *Ae*. *aegypti* in a nutrient-rich, low volume environment. Experiments were conducted at multiple levels of larval density and under both constant and diurnal temperature settings. Data were analyzed to determine the effects of both diurnal temperature and larval density on the development of *Ae*. *aegypti*. Understanding the role of environmental conditions on larval development will provide greater insight into the population dynamics of the *Ae*. *aegypti* populations.

## Methods

### *Ae*. *aegypti* colony

Experiments were conducted using eggs from *Ae*. *aegypti* (Liverpool strain) reared under lab conditions. General colony maintenance was performed at 28°C using larval densities less than 0.1 larvae per mL of water. Adults were supplied with a 10% sucrose solution at all times and offered blood meals (sheep) 3 and 7 days following emergence, with egg collection at 7 and 11 days after emergence, respectively.

### Experiments

**Fig 1:**
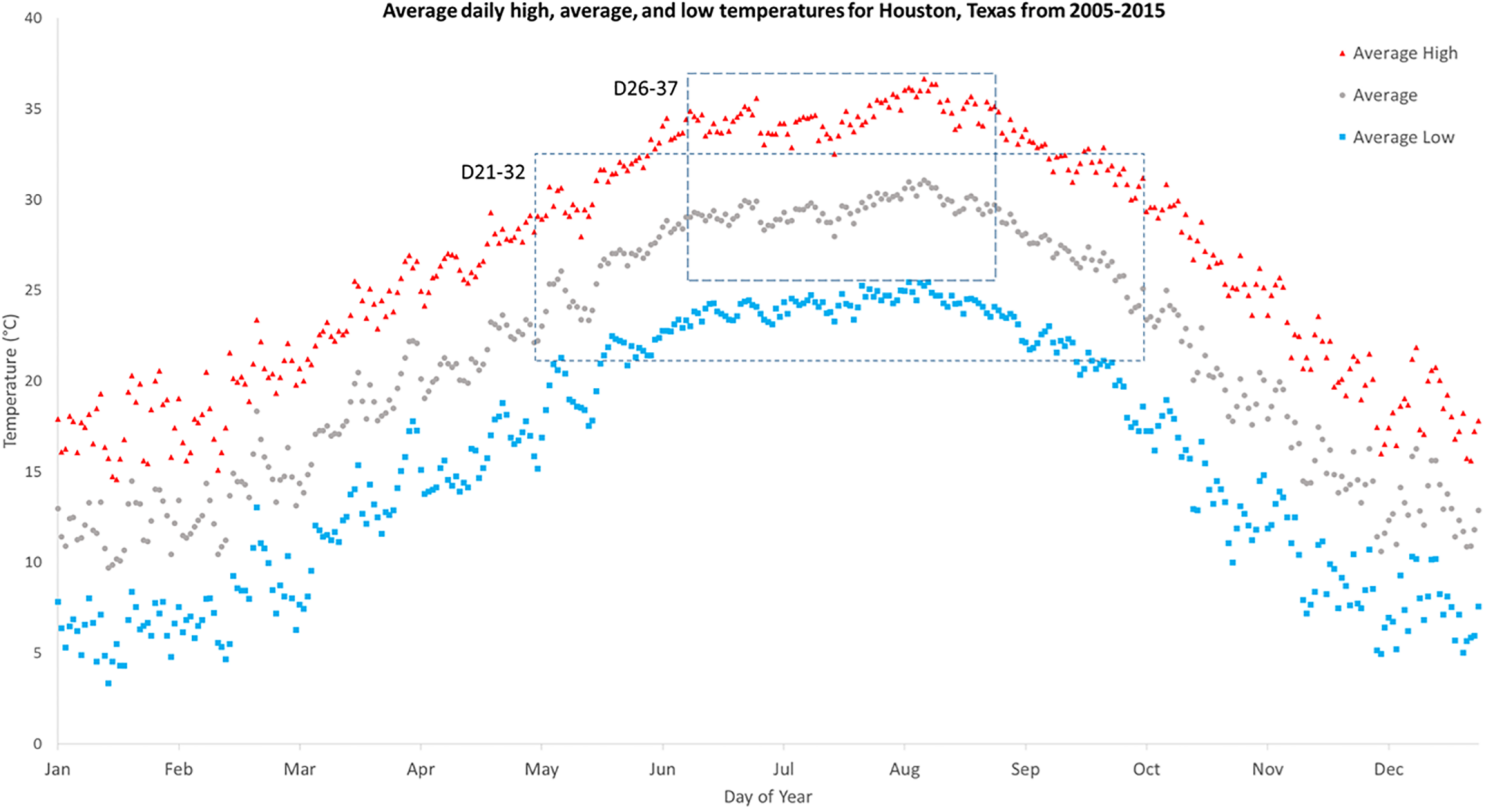
Average daily high, average, and low temperatures for Houston, Texas.

Daily weather data from Jan. 1, 2005 to Dec. 31, 2015 for Houston, Texas was obtained from Weather Underground [30] and analyzed for daily high, average, and low temperature values to obtain the pattern and timing of local temperature fluctuations. Averages of the daily high, average, and low temperatures were obtained for each calendar day (Fig 1). Daily temperature fluctuations averaged 11.1°C, with low temperatures following sunrise and high temperatures peaking in the late afternoon (nine hours after sunrise). Two daily temperature fluctuations were evaluated under laboratory settings. Temperatures varying between 21 and 32°C (abbreviated D21-32), corresponded to environmental conditions from May-June and September-October, while fluctuations between 26.5 and 37.5°C (abbreviated D26-37) corresponded to environmental conditions from June-August.

Diurnal experiments were conducted in Caron refrigerated incubators (model 7001-25-1-039), with temperature varying between 21–32°C and 26.5–37.5°C. Cycling temperatures were set with the low temperature occurring at sunrise (7:00 AM) and increasing linearly for nine hours to the high temperature at mid-afternoon (4:00 PM). Following the peak, temperature was decreased linearly for 15 hours until the subsequent sunrise. Constant temperature experiments were conducted for comparison in Thermo scientific incubated environmental chambers (3960 series) at 26.5°C and 32°C (abbreviated C26 and C32, respectively) as the average temperature settings for D21-32 and D26-37, respectively. Chambers were maintained at 80% relative humidity at all times. Indirect light exposure was set to 12 hours (7:00 AM– 7:00 PM), synchronized to represent the natural conditions.

Cups containing 97.5, 17.5, 7.5, 2.5, and 1.5 ml of water were placed into the incubation chambers at each respective temperature three days prior to beginning experiments to ensure the water reached the temperature of the chambers. An excess amount of water was also placed in each chamber for initial use for vacuum hatching. Cups were covered with lids to limit evaporation and for containment. Each cup was numbered for data collection.

Eggs from *Ae*. *aegypti* were placed into a beaker containing water heated at the respective temperature of each chamber and hatched under vacuum to ensure all larvae started development simultaneously. Within an hour of hatching, water and larvae were gradually poured from the beaker into a petri dish (placed over a dark surface for easier identification of larvae) and 20 larvae were pipetted with 2.5 mL of water into each cup of 97.5, 17.5, 7.5, 2.5, and 1.5 mL of R_0_ (distilled) water to create initial densities of 0.2, 1, 2, 4, and 5 larvae per mL of water, respectively. Pipettes were flushed with water from each cup several times to ensure no larvae were stuck inside the pipette between counts. Each experiment consisted of 45 trials (cups) at a particular larval density and temperature setting for a total of 225 trials within one chamber and a total of 900 trials across all experiments.

Larvae were fed with cichlid fish pellets (each pellet weighing 14.5 mg) to maintain a consistent feeding pattern and to keep the water cleaner than with fish flakes. Feeding for 0.2, 1, and 2 larvae per mL larval densities was set with one pellet upon hatching, and one pellet at 48 and 72 hours after hatching. Feeding for 4 and 5 larvae per mL was set to a half pellet upon hatching, and a half pellet at 36, 48, 60, 72, and 84 hours after hatching to maintain water clarity and limit excessive bacterial growth. Additional feeding outside of this schedule occurred when the amount of food in the containers was minimal. The goal of the feeding schedule was to have a constant source of nutrition for the larvae while maintaining low water turbidity, so that nutrition and excessive bacterial growth were not constraints on larval development.

Since the development of pupae has been shown to be independent of density [31] and to ease with the data collection, pupae were transferred from the initial containers into separate containers. Excluding feeding, pupal containers were maintained under the same conditions as larval containers.

### Data collection

Cups were observed twice daily until fourth instar larvae (L4) were present. At the first appearance of L4 larvae, the number of pupae and adults in the cups was recorded every six hours. When 95% of pupae had emerged, data collection was performed every 12 hours. From the time of the first pupation, numbers of new pupae and dead larvae were recorded for eight days. Following this period, the number of remaining larvae were counted. Likewise, numbers of emerged adults and dead pupae were recorded for eight days from the time of the first adult emergence and the number of remaining pupae were recorded. Experiments were concluded following this period since the majority of remaining larvae would die (more than 95%).

**Fig 2:**
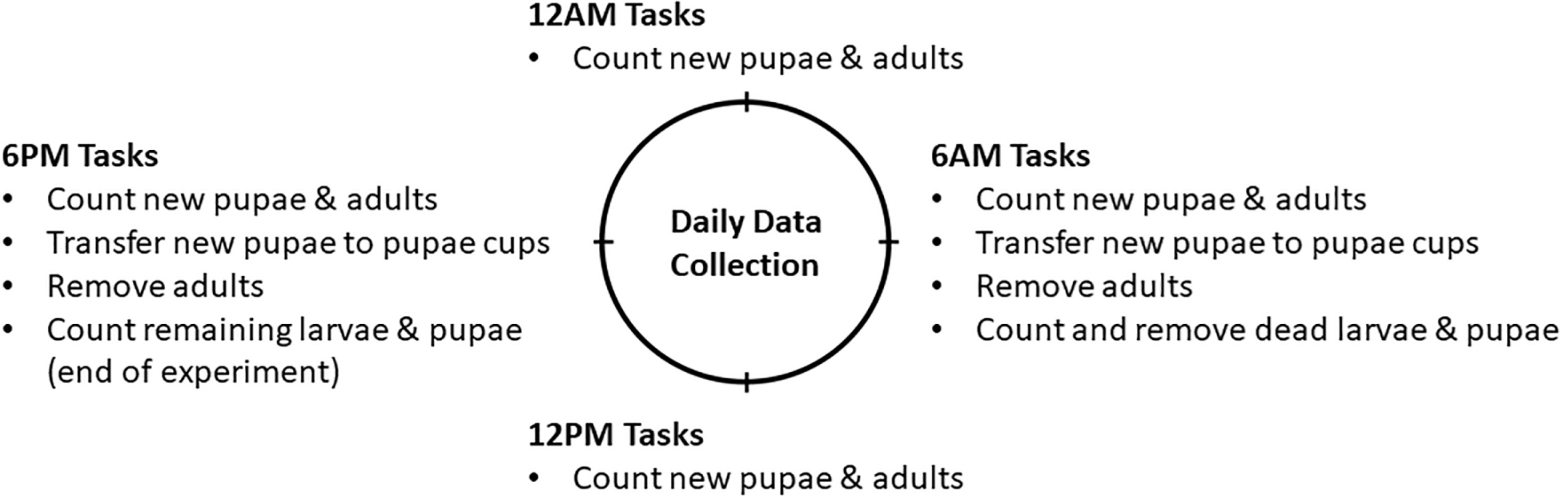
Daily data collection process.

The number of dead larvae and dead pupae was recorded once daily following the first pupation. Larval mortality was not recorded during the time period from hatching to first pupation. Since the number of larvae in each cup was constant (20 larvae per cup), the larval mortality during this time was accounted for by subtracting the number of pupae, dead larvae, and larvae remaining at the end of the data collection from the initial number of larvae allocated at the start of the experiments. Dead larvae, dead pupae, and adult mosquitoes were removed from their respective containers. Upon the completion of the experiments, the number of remaining larvae and pupae was recorded (Fig 2).

### Statistical analysis

All analysis was conducted using MATLAB R2016a (The MathWorks, Inc. 2016) and Minitab 17 (Minitab Inc. 2017). Criteria evaluated consisted of (1) the final proportion of larval mortality, (2) the final proportion of pupal mortality, (3) the time to first pupation, (4) the time to first adult emergence, and (5) the final proportion of adult emergence. The time of first pupation was defined as the first observation of pupae in the container and the time of first emergence was defined as the first time at which at least one adult mosquito was observed.

Residuals from a general linear model of the original data with temperature setting, larval density, and interaction effects from both of these factors were normally distributed with comparable variances. Furthermore, with independent sampling of each trial, all conditions for conducting an analysis of variance (ANOVA) were satisfied. For each of the criteria, a two-factor ANOVA was conducted to identify the effects of temperature settings and larval densities, as well as any effects caused by the interaction of these two factors. The two-factor ANOVA comparisons were made between each diurnal temperature pattern and the respective constant average temperature (D21-32 was compared to C26 while D26-37 was compared to C32). Tukey pairwise comparisons were made between all larval densities at each temperature setting (e.g. under D32, 1 larvae/mL was compared to 2 larvae/mL) and between equal larval densities across constant and diurnal temperatures (e.g. 4 larvae/mL under C26.5 was compared to 4 larvae/mL under D21-32). An average temperature factor (26.5 or 32°C) and was also evaluated across all experiments. A three-factor ANOVA (consisting of average temperature, temperature pattern, and larval density) was conducted for each factor and their interactions for all of the criteria evaluated.

## Results

**Fig 3:**
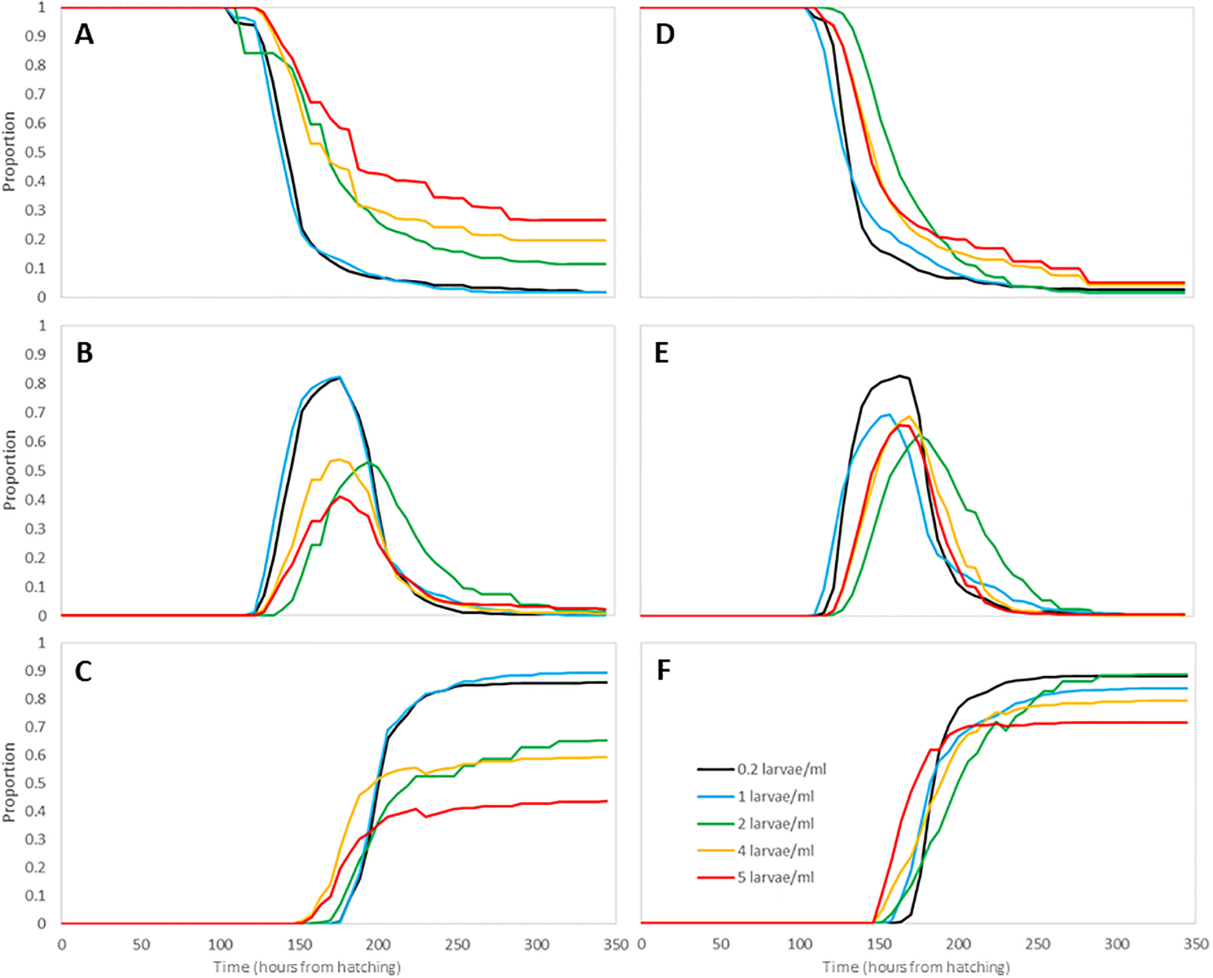
(A) Proportion of larvae under D21-32. (B) Proportion of pupae under D21-32. (C) Proportion of emerged adults under D21-32. (D) Proportion of larvae under C26. (E) Proportion of pupae under C26. (F) Proportion of emerged adult mosquitoes under C26.

**Fig 4:**
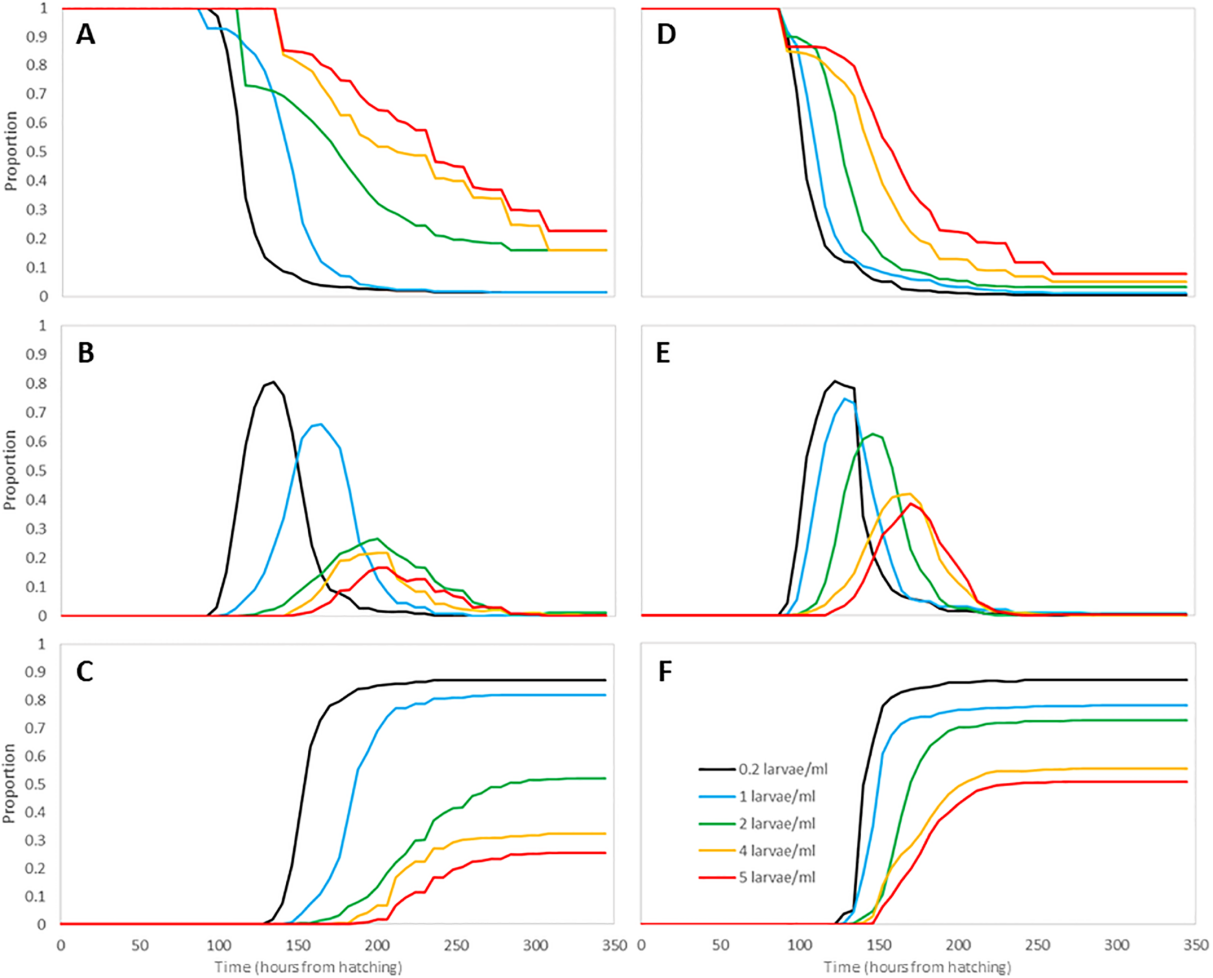
(A) Proportion of larvae under D26-37. (B) Proportion of pupae under D26-37. (C) Proportion of emerged adults under D26-37. (D) Proportion of larvae under C32. (E) Proportion of pupae under C32. (F) Proportion of emerged adult mosquitoes under C32.

In the present study, each criterion was evaluated under both constant and diurnal temperatures across multiple levels of larval density. Figs 3 and 4 show the proportions of larvae, pupae, and emerged adults averaged across all containers for each temperature setting and larval density used in this study.

### Final larval mortality

**Fig 5:**
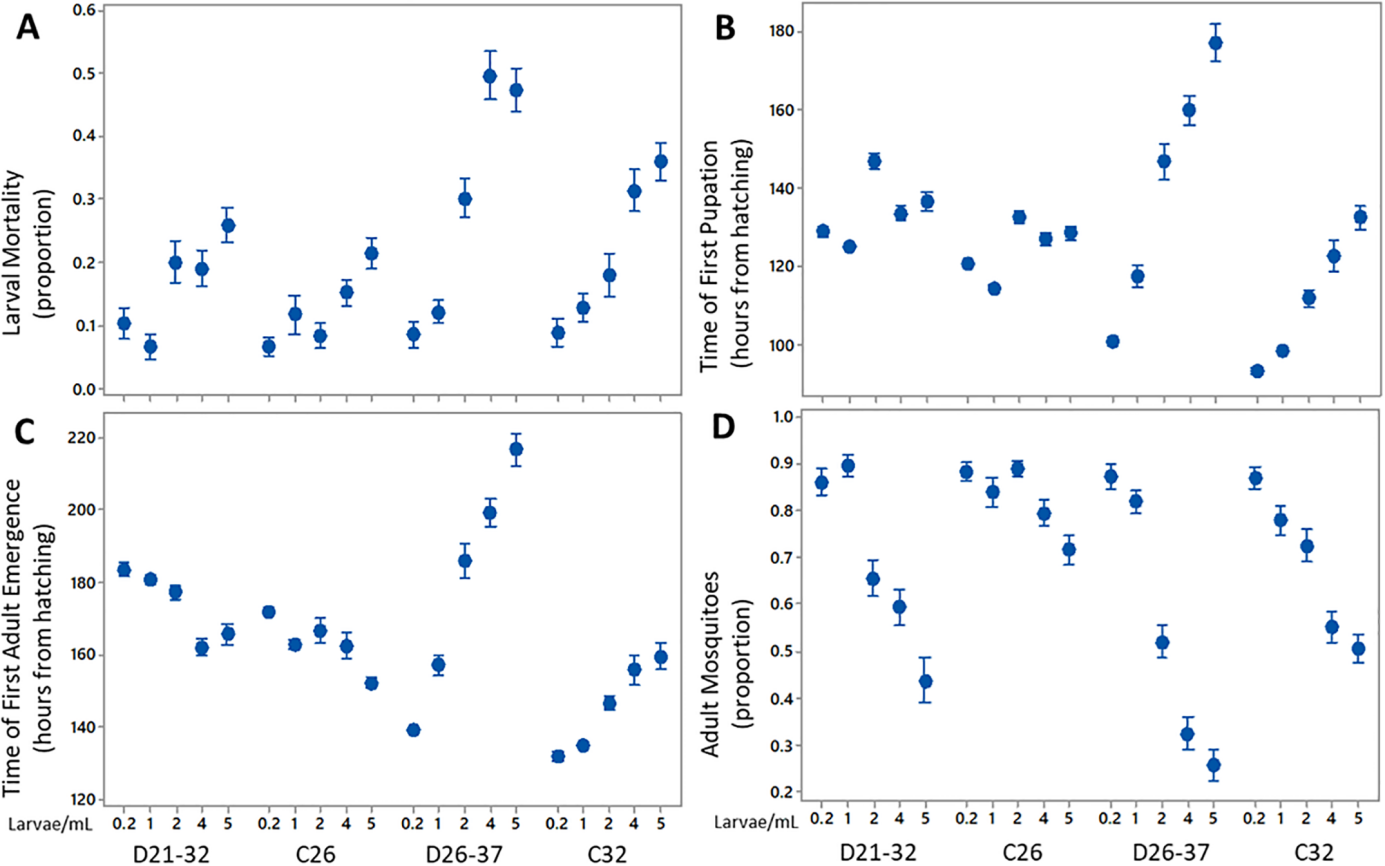
(A) Mean proportion of larval mortality with 95% confidence intervals. (B) Mean time of first pupation with 95% confidence intervals. (C) Mean time of first adult emergence with 95% confidence intervals. (D) Mean proportion of adult mosquitoes with 95% confidence intervals.

Larval mortality was significantly higher at high larval densities under all rearing temperatures and under diurnal temperature patterns when compared to constant temperatures. The mean proportion of larval mortality with 95% confidence intervals is shown in Fig 5A with grouping provided in Table A in S1 Table. Table B in S1 Table details pairwise comparisons of larval densities within each temperature group (e.g. between 1 and 2 larvae/mL under C26). Comparisons between D21-32 and C26 show no significant difference (p>0.05) in larval mortality across temperature settings, under 0.2, 1, 4, and 5 larvae/mL (see Table C in S1 Table). Two-factor ANOVA of larval mortality showed significant differences based on temperature pattern (F = 23.10, p<0.001), larval density (F = 50.19, p<0.001), and the interaction between temperature pattern and larval density (F = 11.60, p<0.001) in D21-32 and C26 experiments (Table A in S2 Table).

Comparing across temperature settings between D21-32 and C32, only the lowest larval densities of 0.2 and 1 larvae/ mL showed no significant difference (p>0.05) in larval mortality, while higher larval densities of 2, 4, and 5 larvae/ mL demonstrated a significant difference (p<0.001) as shown in Table C in S1 Table). Two-factor ANOVA results showed a significant differences due to temperature pattern (F = 77.98, p<0.001), larval density (F = 221.58, p<0.001), and the interaction between temperature pattern and larval density (F = 16.12, p<0.001) within D26-37 and C32 experiments (Table B in S2 Table).

**Fig 6:**
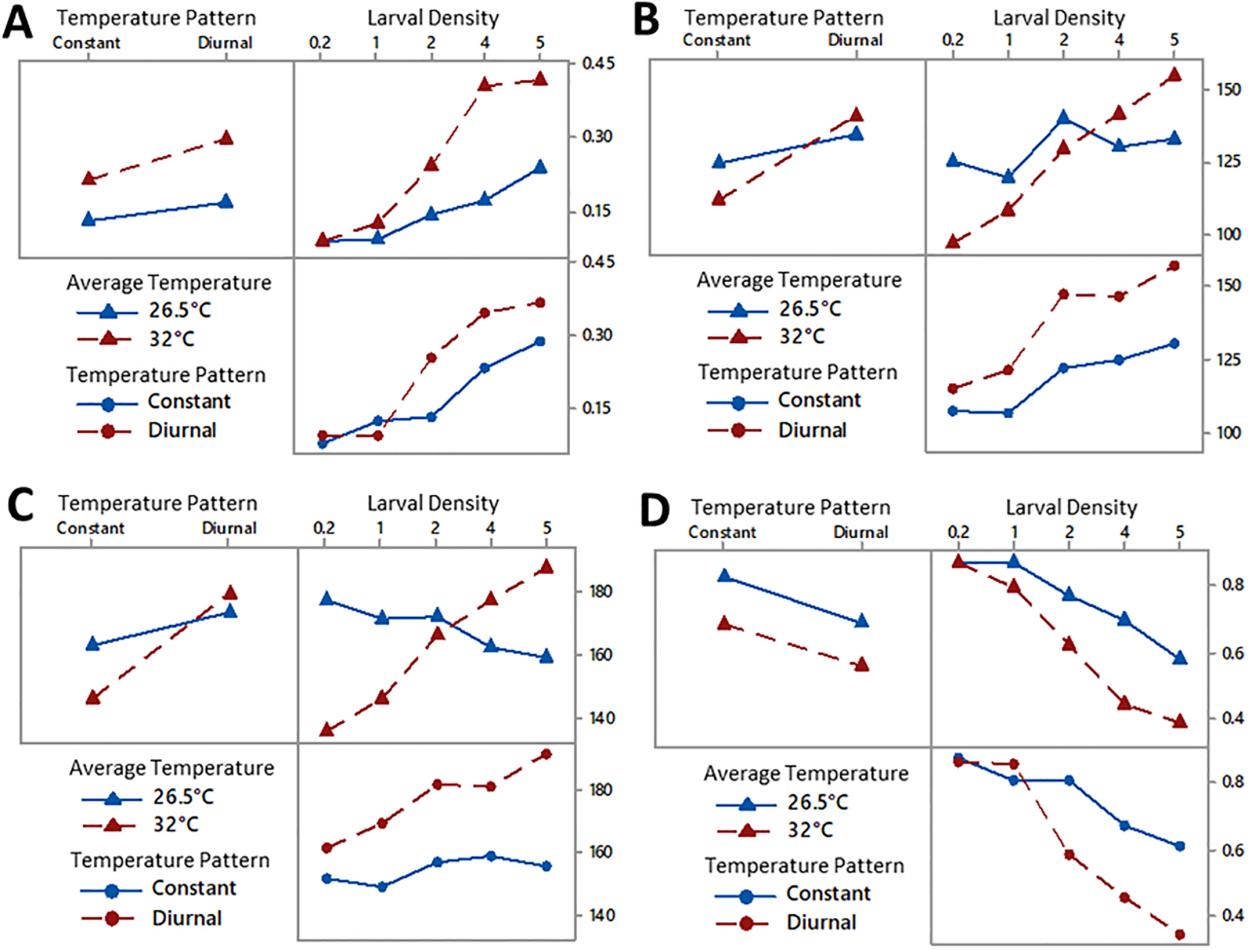
(A) Interaction plot of proportion of larval mortality. (B) Interaction plot of first pupation. (C) Interaction plot of first adult emergence. (D) Interaction plot of proportion of adult mosquitoes.

Analysis of the three-factor ANOVA (shown in Table A in S3 Table) showed significant differences based on the average temperature (F = 328.38, p<0.001), temperature pattern (F = 96.83, p<0.001), and larval density (F = 247.27, p<0.001). Furthermore, the two-factor interactions of average temperature and temperature pattern (F = 13.04, p<0.001), average temperature and larval density (F = 51.92, p<0.001), and temperature pattern and larval density (F = 21.84, p<0.001) were shown to be significant. The three-factor interaction of average temperature, temperature pattern, and larval density was significant (F = 6.06, p<0.001). Interaction plots of the proportion of larval mortality is shown in Fig 6A.

### Time of first pupation

The time to the first observed pupation was shorter when mosquitoes were reared under constant temperatures than when the mosquitoes were reared at diurnal temperatures, and this was true at each of the larval densities tested. Mean times to the first observed pupation with 95% confidence intervals is shown in Fig 5B, while grouping of the results is detailed in Table D in S1 Table. Pairwise comparisons of the time to first pupation under D21-32 indicate a significant difference (each with p<0.05) between all larval densities, except between the two highest larval densities (p = 0.203) as shown in Table E in S1 Table. Likewise, under C26, only the difference between 4 and 5 larvae/mL was not significant (p = 0.905). A pairwise comparison between equal larval densities across D21-32 and C26 shows a significant difference (each with p<0.001) in the time to the first observed pupation (Table F in S1 Table). Two-factor ANOVA (shown in Table C in S2 Table) on the time to first pupation for D21-32 and C26 experiments determined significant differences due to temperature pattern (F = 356.17, p<0.001), larval density (F = 183.48. p<0.001), and the interaction between temperature pattern and larval density (F = 7.23, p<0.001).

Pairwise comparisons of larval densities under D26-37 show a significant difference (p<0.001) for all combination of larval densities (see Table D in S1 Table). Under C32, pairwise comparisons of larval densities indicate no significant difference (p = 0.322) between 0.2 and 1 larvae/mL, while all other comparisons showed a significant difference (p<0.001) as shown in Table E in S1 Table. Pairwise comparisons with equal larval densities across temperature settings of D26-37 and C32 show a significant difference (p<0.05) in time to first pupation across all larval densities (Table F in S1 Table). Furthermore, an amplification of differences (increasing t-values) between D26-37 and C32 appears as larval density increases. Two-factor ANOVA results show significant differences due to temperature pattern (F = 860.89, p <0.001), larval density (F = 467.94, p<0.001), and the interaction between temperature pattern and larval density (F = 46.96, p <0.001) for the D26-37 and C32 experiments (see Table D in S2 Table).

The three-factor ANOVA (Table B in S3 Table) showed that all combinations of factors and interactions were significant. Average temperature (F = 37.91, p<0.001), temperature pattern (F = 1205.71, p<0.001), and larval density (F = 554.69, p<0.001) had a significant impact on the time to first pupation. The two-factor interactions of average temperature and temperature pattern (F = 305.06, p<0.001), average temperature and larval density (F = 262.35, p<0.001), and temperature pattern and larval density (F = 38.81, p<0.001) were also shown to be significant. The interaction plot of the time to first pupation is shown in Fig 6B. Finally, the three-factor interaction of average temperature, temperature pattern, and larval density was also significant (F = 38.50, p<0.001).

### Time to emergence of first adults

The mean time to the first adult emergence and grouping of results is summarized in Table G in S1 Table. The mean times are also plotted in Fig 5C with 95% confidence intervals. In pairwise comparisons between larval densities under D21-32, differences between 0.2 and 1, 1 and 2, and 4 and 5 larvae/mL were found to be not significant (each with p>0.05). All other pairwise comparisons of time to the first adult emergence between larval densities at D21-32 were significant (each with p<0.05) as shown in Table H in S1 Table. Pairwise comparisons of larval densities under C26 revealed differences between 1 and 2, 1 and 4, and 2 and 4 larvae/mL were not significant (p>0.05), while all remaining comparisons showed a significant difference (each with p<0.05). Comparisons under the same larval density between D21-32 and C26 showed significant differences (each with p<0.001) across all larval densities, except under 4 larvae/mL (see Table I in S1 Table). Two-factor ANOVA shows a significant difference in the time to first adult emergence due to temperature pattern (F = 219.87, p<0.001), larval density (F = 92.56, p<0.001), and the interaction between temperature pattern and larval density (F = 18.01, p<0.001) within the D21-32 and C26 experiments (Table E in S2 Table).

Under D26-37, pairwise comparisons of time to first adult emergence between larval densities determined a significant difference (each with p<0.001) among all pairs (see Table H in S1 Table). Pairwise comparisons of larval density under C32 showed no significant difference (each with p>0.05) between 0.2 and 1, and 4 and 5 larvae/mL. All other comparisons were significant (each with p<0.05) under C32. Comparisons of equal larval densities across D26-37 and C32 showed a significant difference (p<0.05) at all levels, with an amplification in differences (increasing t-values) as larval density increased (see Table I in S1 Table). Within the D26-37 and C32 experiments, two-factor ANOVA (shown in Table F in S2 Table) reveals significant differences due to temperature pattern (F = 1095.07, p<0.001), larval density (F = 359.62, p<0.001), and the interaction between temperature pattern and larval density (F = 71.49, p<0.001).

Further analysis with the three-factor ANOVA (shown in Table C in S3 Table) revealed the individual factors of average temperature (F = 86.30, p<0.001), temperature pattern (F = 1268.37, p<0.001), and larval density (F = 111.45, p<0.001) to have a significant impact on the time to emergence of the first adult mosquitoes. Two-factor interactions of average temperature and temperature pattern (F = 346.37, p<0.001), average temperature and larval density (F = 432.22, p<0.001), and temperature pattern and larval density (F = 44.28, p<0.001) also had a significant impact. Additionally, three-factor interactions of average temperature, temperature pattern and larval density had a significant impact on the time of the first emergence of adult mosquitoes. The interaction plot of the time to first adult emergence is shown in Fig 6C.

### Final proportion of adults

The proportion of adults in relation to the total number of mosquitoes at all life-stages was determined at the end of each experiment. Mean and grouping of the final proportions of adults is provided in Table J in S1 Table. Furthermore, final proportions of adults with 95% confidence intervals are shown in Fig 5D. Pairwise comparisons according to larval density under D21-32 shows no significant difference between 0.2 and 1, and 2 and 4 larvae/mL (each with p>0.05). All remaining comparisons show a significant difference (p<0.001) in the final proportion of adults across all larval densities compared (see Table K in S1 Table). Under C26, pairwise comparisons across larval densities shows no significant differences between 0.2 and 1, 0.2 and 2, 1 and 2, and 1 and 4 larvae/mL (each with p>0.05), while all other combinations show significant differences (each with p<0.05). Comparisons across D21-32 and C26 with equal larval densities shows no significant difference at 0.2 and 1 larvae/mL (with p = 0.987 and p = 0.219, respectively) and a significant differences at 2, 4, and 5 larvae/mL (each with p<0.001) as shown in Table L in S1 Table. Two-factor ANOVA results show that there is a significant difference in the final proportion of adult mosquitoes due to the temperature pattern (F = 191.00, p<0.001), larval density (F = 127.86, p<0.001) and the interaction of temperature pattern and larval density (F = 43.43, p<0.001) within the D21-32 and C26 experiments (see Table G in S2 Table).

Pairwise comparisons conducted on the data for D26-37 shows a significant difference (each with p<0.05) between all larval densities except between 0.2 and 1 larvae/mL (p = 0.258) as shown in Table K in S1 Table. Under C32, pairwise comparisons across larval densities shows a significant difference (p<0.05) across all pairs except between 1 and 2, and 4 and 5 larvae/mL (with p = 0.270 and p = 0.487, respectively). Comparisons across D26-37 and C32 with equal larval densities show no significant difference at the 0.2 and 1 larvae/mL levels (with p = 1.000 and p = 0.711, respectively) and a significant difference at the 2, 4, and 5 larvae/mL levels (see Table L in S1 Table). The two-factor ANOVA results (shown in Table H in S2 Table) indicated a significant difference in the final proportion of adult mosquitoes due to temperature pattern (F = 174.66, p<0.001), larval density (F = 394.42, p<0.001), and the interaction between temperature pattern and larval density (F = 40.72, p<0.001) for the D26-37 and C32 experiments.

Analysis of the data with a three-factor ANOVA showed average temperature (F = 376.82, p<0.001), temperature pattern (F = 365.60, p<0.001), and larval density (474.80, p<0.001) had a significant impact on the final proportion of adult mosquitoes. The interaction plot of proportion of adult mosquitoes is shown in Fig 6D. Additionally, the two-factor interaction of average temperature and larval density (F = 42.64, p<0.001), and temperature pattern and larval density (F = 83.36, p<0.001) were significant. The two-factor interaction of average temperature and temperature pattern (F = 0.36, p = 0.548) and the three-factor interaction of average temperature, temperature pattern, and larval density (F = 0.85, p = 0.497) did not have a significant impact on the final proportion of adult mosquitoes (See Table D in S3 Table).

## Discussion

The goal of this study was to analyze the combined impact of diurnal temperature fluctuations and larval density on the development of *Ae*. *aegypti*. By controlling the amount of water that larvae developed in, we created different larval densities to study the effects of these conditions on mosquito development. We analyzed baseline environmental data to assess the daily temperature fluctuations mosquitoes might be exposed to and applied these settings across all larval densities. For comparison, we conducted experiments at constant temperatures commonly used in laboratory experiments in order to identify any developmental differences that might exist under these conditions.

These results can play a significant role in the estimation of wild *Ae*. *aegypti* populations, specifically under high temperatures. Compared to the experiments conducted at constant temperatures, the results show that diurnal temperatures have a significant impact on the number of adults that enter a population. These results may prove to be specifically useful to areas with high average temperatures (greater than 32°C). Although the difference is smaller, results obtained under lower temperatures also indicate significant differences between constant and diurnal temperatures.

To address the factors of temperature pattern and larval density, we controlled other factors that also play an important role in the development of *Ae*. *aegypti*. To limit competition between larvae for nutrients, an unlimited high protein diet was provided, which is likely not the case in natural environmental conditions. This allowed us to focus solely on the impacts of temperature and larval density, however, such conditions are not likely to be present outside of the laboratory. Additionally, a laboratory strain of *Ae*. *aegypti* was used, which may not fully resemble *Ae*. *aegypti* in nature. Due to the laboratory settings at which experiments were conducted, sunlight exposure, evaporation, and precipitation were further limitations of this study.

Evaluating equal larval densities impacts across temperature patterns, we noticed no significant difference in larval mortality across all larval densities (excluding 4 larvae/mL) in the D21-32 and C26 experimental groups. However, evaluating the larval mortality using the same criteria in the D26-37 and C32 experimental groups, we noticed significant differences among the three highest larval densities (Table C in S1 Table). While not present in the comparison between D21-32 and C26, this significant difference among high larval densities between D26-37 and C32 suggests an amplification of the difference under higher temperature settings. Additionally, the mean proportions of larval mortality among the three highest larval density experiments was higher under diurnal temperature setting when compared to the respective constant temperatures. The increasing proportion of larval mortality with increasing larval density suggests that the larval mortality increases under high larval densities, especially under diurnal temperature patterns. Our results were comparable to those presented by Yang et al. (2009) and Mohammed et al. (2011) for our lower larval density experiments [12,27,32].

Evaluation of the first time of pupation in the pairwise comparison conducted across all temperature patterns with equal larval densities showed a significant differences due to temperature pattern (Table F in S1 Table). Additionally, the mean time to first pupation under constant temperature patterns was shorter than the time under the respective diurnal temperature and larval density. The increased time to the first pupation as larval density was increased and subsequent longer time under diurnal temperature patterns support the claim of delayed pupation in highly dense larvae containers under diurnal temperature patterns when compared to low larval density containers under constant temperatures.

Within the D26-37 and C32 experiments, there were significant differences in the time of first pupation across all larval densities when compared across temperature settings. Carrington et al. (2013) reports on the *Ae*. *aegypti* aquatic stage rate of development as increasing up to 35°C and decreasing beyond this threshold. Since the experiments we conducted under D26-37 exceeded this threshold, it is likely that the prolonged time to the first adult emergence was a result of the time the juvenile mosquitoes spent above 35°C. Furthermore, increasing t-values (shown in Table A6 in S1 Table) demonstrate an increasing difference in time to first pupation due to temperature pattern between the D26-37 and C32 as larval density increases. This suggests that the time to the first pupation is impacted more significantly under the combination of high larval density and high diurnal temperature fluctuations. The results we obtained were comparable to those presented in Carrington et al. (2013) [32].

The one-, two-, and three-factor interactions from the three-factor ANOVA conducted showed that there was a significant difference (each p<0.001) due to the combinations of average temperature, temperature pattern, and larval density. These results show that the larval mortality and the time of the first pupation is dependent on the combinations of the three factors examined.

Based on the results presented for the time to first adult emergence under D21-32 and C26, there was a significant difference between equal larval densities across these two temperature settings. Only one of the larval densities (4 larvae/mL) did not show a significant difference between temperature patterns in these experiments. This is likely due to an error that occurred in data collection, as the remainder of the larval densities were consistently significant. Likewise, comparisons conducted between the D26-37 and C32 temperature patterns with equal larval densities showed significant differences at all levels. As with the time to first pupation, t-values follow an increasing trend with increases in larval density. This supports the claim that aquatic development of *Ae*. *aegypti* is more significantly impacted under high larval densities at high diurnal temperatures that pass the 35°C threshold. To our knowledge, there have been no other similar studies that have examined the time to the first adult emergence.

Comparison of the final proportion of adult mosquitoes between temperatures shows an increasing trend between both diurnal temperature patterns and the respective constant temperatures when compared with equal larval densities as larval density increases. In both comparisons, there was no significant difference between constant and diurnal temperature patterns at the lowest larval densities of 0.2 and 1 larvae/mL. However, as the larval density was increased, the difference between the constant and diurnal temperatures increased. The results also show a decreasing trend in the final proportion of adults decreasing as larval density increases across all temperature settings.

Furthermore, the mean proportion of adult mosquitoes is smaller under the higher temperature patterns (D26-37 and C32) when compared to the lower temperature patterns (D21-32 and C26) at larval densities higher than 0.2 larvae/mL. Conversely, the time of the first adult emergence is shorter for D26-37 and C32 when compared to D21-32 and C26. As shown by Carrington and Yang, this demonstrates the aquatic development occurs faster under higher temperatures, but the overall number adult mosquitoes decreases with increases in temperature [32,33]. Unfortunately, we did not run analyses on the rates of adult emergence, but based on the final proportion of adults and time to first adult emergence, we expect these rates to be inversely correlated with increases in larval density at diurnal temperature fluctuations.

The one-, two, and three-factor interactions show a significant difference (p<0.001) in the time to the first adult emergence due to the average temperature, temperature pattern, and larval density for all combinations of these factors. However, while the final proportion of adults was significantly impacted by each factor individually, only the two-factor interaction between average temperature and larval density caused a significant difference (p<0.001). The remaining two-factor interaction of average temperature and temperature pattern, and the temperature pattern and larval density across all experiments were not significant (p>0.05). Furthermore, the three-factor interaction also did not have a significant impact (p>0.05) on the final proportion of adult mosquitoes. These results show each of the factors has an individual impact on the final proportion of adults, but within the two- and three-factor interactions, temperature pattern does not have an interacting effect on the final proportion of adult mosquitoes.

## Conclusion

*Ae*. *aegypti* continues to be one of the leading vectors in the spread of arboviruses in many parts of the world [34–37]. Understanding the impact of environmental conditions on the development of these vectors can provide insight into the population dynamics of these vectors and can allow for prediction of disease incidence within humans and domestic animals. The results presented in this paper show that the combined effects of diurnal temperature and larval densities can significantly impact the time, rate, and overall proportion of pupae and emerging adults within a breeding site. Vector population modeling and epidemiological analysis should factor both diurnal temperature and density to improve the accuracy of population estimates.

Our results show that larval density and diurnal temperature can significantly impact the development of *Ae*. *aegypti* and that there are significant interaction effects between these factors in relation to the development parameters analyzed in this study. It can be concluded that mosquito population models that use experimental data conducted under constant temperatures and do not account for larval density as a parameter may be over- or under-estimating the population of adult mosquitoes in an area. While there are other factors that must be addressed within these population models, the use of larval density and diurnal temperature can give a more accurate representation of the mosquito population.

Many efforts to model wild mosquito populations have overlooked the importance of larval density [38–43]. Furthermore, these models attempt to model mosquito populations according to experimental data conducted under constant temperature and low larval density. While these models do limit the population growth with a carrying capacity, the rates at which pupation occurs is impacted both by the temperature range and the larval density. Without addressing these factors within the models, the adult mosquito populations and the disease spread may be drastically over- or under-estimated.

Our future research will focus on modeling the growth of *Ae*. *aegypti* using mathematical and machine learning models containing diurnal temperatures and larval densities as explanatory variables. We also plan to study the micro-climatic conditions in the breeding and nesting locations and develop mathematical models for *Ae*. *aegypti* development and their susceptibility to Zika virus.

## Supporting information

S1 Table Results from Tukey pairwise comparisons. (XLSX)

S2 Table Results from two-factor ANOVA. (XLSX)

S3 Table Results from three-factor ANOVA. (XLSX)

S4 Table Dataset of experimental values used in ANOVA calculations. (XLSX)
